# Checklist and key for the identification of fish fauna of the Uberaba River, Upper Paraná River system, Brazil

**DOI:** 10.3897/zookeys.875.31977

**Published:** 2019-09-16

**Authors:** Douglas de Castro Ribeiro, Jumma Miranda Araújo Chagas, Mariana Ribeiro Thereza, Francisco Langeani

**Affiliations:** 1 UNESP, Universidade Estadual Paulista, Instituto de Biociências, Letras e Ciências Exatas, Departamento de Zoologia e Botânica, Laboratório de Ictiologia, Rua Cristóvão Colombo, 2265, 15054-000 São José do Rio Preto, SP, Brazil Universidade Estadual Paulista Rua Cristóvão Colombo Brazil; 2 UNESP, Universidade Estadual Paulista, Instituto de Biociências, Departamento de Biologia e Zootecnia, Laboratório de Ecologia do Parasitismo, Ilha Solteira, SP, Brazil Universidade Estadual Paulista Ilha Solteira Brazil

**Keywords:** Brazilian Cerrado, freshwater fish, Neotropical Region, rheophilic environment, threatened species

## Abstract

The Uberaba River is an important right-bank tributary to the Grande River, in the Upper Paraná River system, Brazil, and the main water source for the public supply of the Uberaba city, Minas Gerais state. An inventory, an identification key, and photographs of the fish species of the Uberaba River are provided, based on samples made between 2012 and 2014 at 14 sampling sites in the river system. A total of 73 species was recorded from six orders, 20 families, and 49 genera. Characiformes and Siluriformes are the most speciose orders and Characidae and Loricariidae are the most commonly recorded families. Most species are autochthonous, nine are considered allochthonous, and two species are exotic. The Uberaba River has a diverse and heterogeneous ichthyofauna, typical of rheophilic environments, with endemic species and few non-native species.

## Introduction

Approximately 34,797 species of fish have been formally described worldwide (Fricke et al. 2018), and recent estimates suggest that ca. 13,000 species are partially or exclusively freshwater (Nelson 2016). The Neotropical region has a unique and diverse freshwater fish fauna (Albert and Reis 2011), with 9,100 species exclusively distributed in South America (Reis et al. 2016), an impressive number when compared to the global estimates. Approximately 43% of the Neotropical fish diversity occurs in Brazil (Buckup et al. 2007), and the Amazon and La Plata river drainages bear the largest fish diversity in South America (Langeani et al. 2007).

With geological origin dating from the Mesozoic (Neocretaceous), the La Plata River has an estimated drainage area of ca. 3 million km² across five countries, Bolivia, Brazil, Paraguay, Argentina, and Uruguay, and is the second largest drainage in South America, with the main drainages the Paraná-Paraguay drainage and Uruguay River (Albert and Reis 2011). The Upper Paraná River system is a catchment above the Sete Quedas Falls, currently flooded by the Itaipu hydroelectric dam, located at the border between Brazil, Paraguay, and Argentina. In the Brazilian portion, the Upper Paraná River system drains the states of Goiás, Minas Gerais, São Paulo, Mato Grosso do Sul, and Paraná, comprising the subsystems of the Grande, Paranaíba, Tietê, and Paranapanema rivers (Souza-Filho and Stevaux 1997; Langeani et al. 2007).

The Upper Paraná River, according to Langeani et al. (2007), harbors approximately 360 of fish species. Subsequently, Fagundes et al. (2015) provide 46 new records for this system. Additionally, at least 28 new species have been described since the last twenty years (e.g., Silveira et al. 2008; Martins and Langeani 2011a, 2011b; Carvalho and Langeani 2013; Serra and Langeani 2015). The increased number of species recorded in the Upper Paraná River in the last decade reflects intense sampling carried out in the region. Some authors (e.g., Langeani et al. 2007; Oyakawa and Menezes 2011) report that the Upper Paraná River is among the most well-sampled Brazilian regions, especially the São Paulo state (Oyakawa and Menezes 2011), and is one of the most impacted by dams, which considerably altered the hydrological regime and natural environments, affecting the dynamics and recruitment in fish populations (Agostinho et al. 2004). Fagundes et al. (2015) carried out intense samplings in tributaries of the Paranaíba, Araguari, and Grande rivers in the state of Minas Gerais, northwest, east, and southeast of the Triângulo Mineiro region, contributing significantly to the knowledge on local fish faunas. However, despite the recent contributions to the Upper Paraná River system, some areas were poorly sampled (e.g., south and southwest of the Triângulo Mineiro region, northeast and south parts of the Minas Gerais state, most of the Mato Grosso do Sul and Goiás states) and information on fish fauna composition and distribution is still missing.

The Uberaba River is a right-bank tributary of the Grande River, in the Upper Paraná River system, Brazil, and it is the main water source for Uberaba city in Minas Gerais state. In the driest period, the water level of the Uberaba River is very low and it is not able to be the only source of public water supply to the Uberaba city. This problem becomes worse with the intensive anthropogenic impact on the environment which results in modifications of hydrological dynamics and associated biotic structures (Candido et al. 2010; Cruz 2003; Valera et al. 2016). A dam located in the middle section of the Uberaba River, designed to capture and treat water for human consumption, significantly altered the natural characteristics and self-depuration capacity of the river (Sousa et al. 2016), even more aggravated by the high loads of raw sewage released into some river sections (Cruz 2003).

The fish fauna of the Uberaba River is only partially known, with only few sections sampled and no seasonal investigations (see SEMEA 2004; Souza et al. 2016). In this paper, we present an inventory of the fish fauna of the Uberaba River based on samples from several sections of the river system. In addition, an identification key and photographs of some species are presented.

## Materials and methods

### Study area

**Figure 1.**
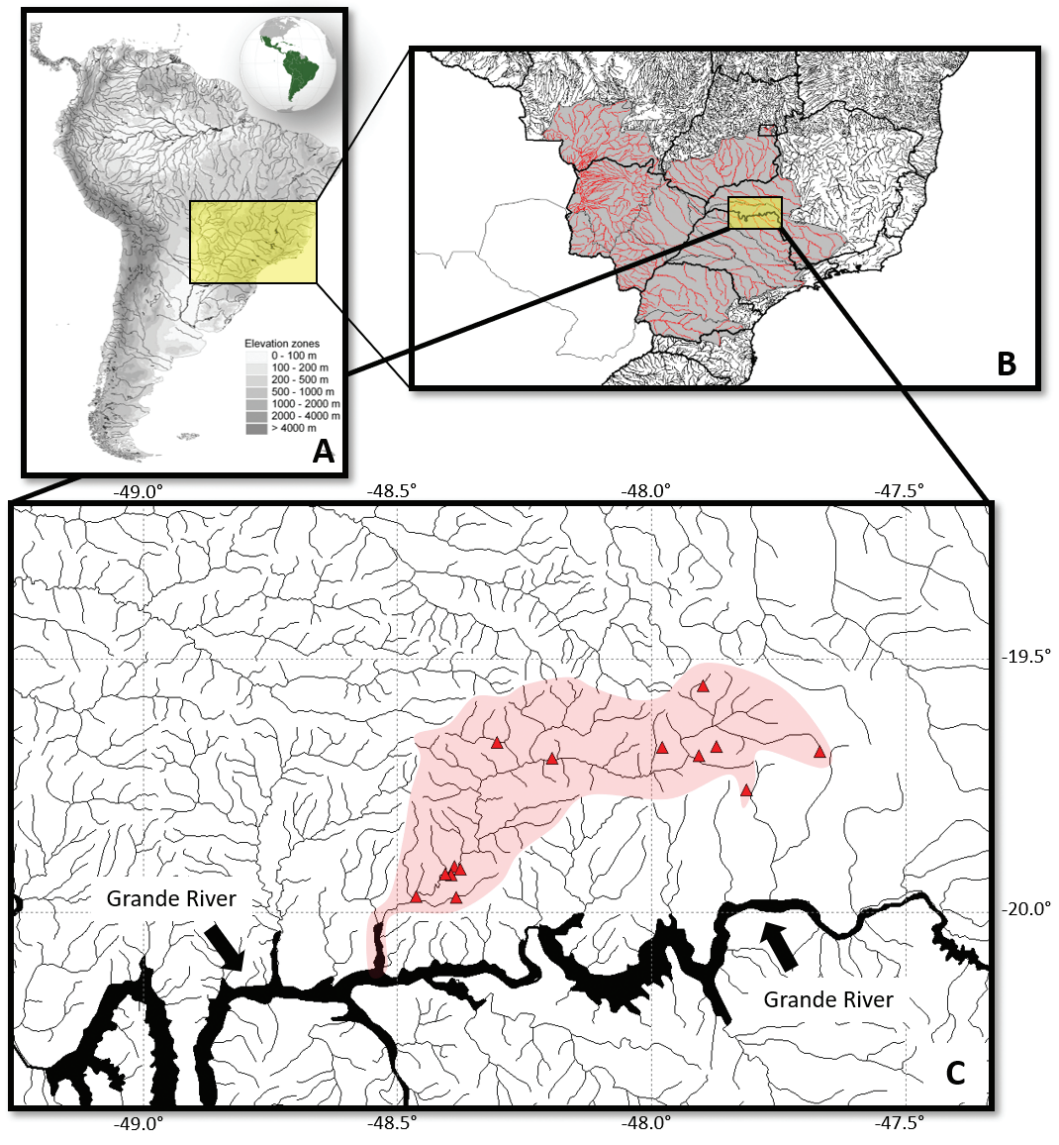
Map of the Uberaba River drainage. **A** Upper Paraná River system highlighted in the Neotropical region **B** location of the Uberaba River drainage in the Upper Paraná River system **C** red triangles showing the sampling sites in the Uberaba River.

The Uberaba River catchment area is located in the southeastern region of Minas Gerais state, Brazil, center-south of the Triângulo Mineiro region, 19°30'37"S – 20°07'40"S; 47°39'2"W – 48°34'34"W (Figure 1). The Uberaba River system covers an area of approximately 2, 428.73 km² and is subordinated to the “Comitê da Bacia Hidrográfica dos Afluentes Mineiros do Baixo Rio Grande (CBH-GD)”. The Uberaba River extends for 184.90 km, with a gap of approximately 554 m, and is supplied by 86 tributaries of diverse orders along its course. Its headwaters are located east of the municipality of Uberaba-MG, a hydromorphic field along the BR-262 road, at 1,014 m of altitude. The Uberaba River discharges in the right side of the Grande River in the municipality of Planura, Minas Gerais state, at 460 m of altitude (CODAU 2005). Along its route, the Uberaba River crosses five municipalities, Uberaba (1,198.75 km²), followed by Conceição das Alagoas (643.19 km²), Veríssimo (568.65 km²), Planura (33.39 km²), and Campo Florido (4.59 km²) (IGAM 2010).

The average annual precipitation in the region ranges between 1,300 mm and 1,700 mm, characterized by a rainy period of six to seven months (October to March) and the driest period (April to September) with less than 60 mm. The thermal regime is defined by an average annual temperature ranging from 20 to 24° Celsius, with a minimum of 18° C in colder months (June/July). These climatic factors characterize two major seasons in the region, one, cold and dry, between autumn and winter, and the other, hot and rainy, between spring and summer (Gomes et al. 1982).

### Data

**Figure 2.**
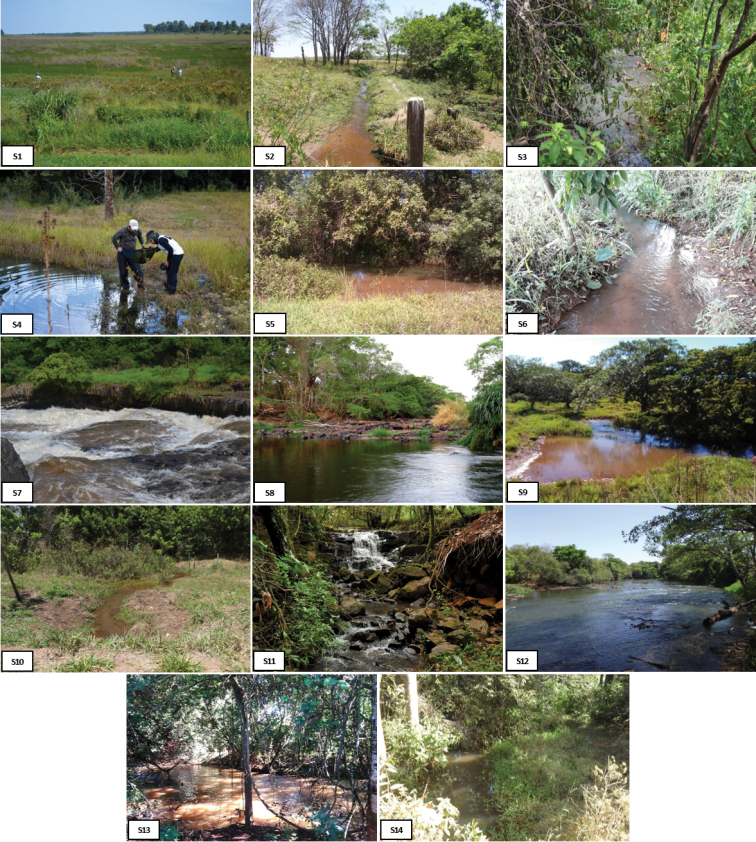
Sampling sites in the Uberaba River, Upper Paraná River system, Brazil. Detailed description of sites in Table 1.

The collections were carried out between 2012 and 2014 in 14 sampling sites (Figures 1, 2; Table 1) along the entire system. Permission for collecting was provided by IEF / DPBIO / GPFF No.44551-1156-2011. The samplings were performed both during the daytime and nighttime, using gill nets (2.5 to 120 mm mesh), dip nets (0.5 mm mesh), seines (1.5 mm mesh), and cast nets (2.5 to 100 mm mesh sizes). After sampling, the specimens were anesthetized in a solution containing 100 mg of eugenol by L^-1^ previously dissolved in 100% ethanol in proportion of 1:1 v/v, fixed in 10% formalin buffered with sodium phosphate (pH 7.0 and 0.2 Mol) for 24 to 72 hours, and then transferred to 70^o^ G.L. ethanol.

**Table 1.** Description of sampling sites (S1 to S14) of the Uberaba River, Upper Paraná River system, Brazil.

Site	Locality	Coordinates	Elevation	Characteristics
S1	Serra do Grotão, headspring of the Uberaba River, on the margins of BR 262, Ponte Alta, MG	19.40575S, 47.405430W	1015	Lentic environment; organic sediment and sand as substrate; clear and warm water, 1 m deep; abundant aquatic plants
S2	Small stream (no name), unpaved road at BR262, tributary of Veríssimo River, Veríssimo, MG	19.39538S; 48.181390W	622	Lotic environment, medium flow; clay as substrate; shallow water, less than 80 cm deep; few marginal plants
S3	Small stream (no name), unpaved road at Mula Preta farm, tributary of the Lageado River, Uberaba, MG	19.45312S; 47.484494W	715	Medium flow stream; sand and clay as substrate; turbid water; less than 1.5 m deep; riparian vegetation and open areas
S4	Small stream (no name), into APP Vale encantado, tributary of the Saudade stream, Uberaba, MG	19.33573S; 47.534852W	901	Lentic environment; organic sediment and sand as substrate; clear and warm water; 0.5 m depth; few aquatic plants
S5	Alegria stream, unpaved road at Alegria farm, tributary of the Uberaba River, Uberaba, MG	19.40224S; 47.522022W	803	Lotic environment, medium flow; clay soil as a substrate; shallow and turbid water, 1 m depth; dense riparian forest and pasture area
S6	Small stream (no name), Rocinha farm, unpaved road at Pará Pereira Gomes road, tributary of the Lageado stream, Uberaba, MG	19.41135S; 47.542032W	778	Lotic environment, medium flow; sand and leaves as substrate; shallow and crystalline waters, 30 cm deep; riparian forest sparse
S7	Uberaba River, below of the PCH Monjolo, Veríssimo, MG	19.41466S; 48.113035W	632	Lotic environment, fast flowing, several rapids and small backwaters, basaltic rocks and sand as substrate, riparian vegetation well preserved.
S8	Uberaba River, Conceição das Alagoas, MG	19.54288S; 48.23155W	495	Lotic environment, fast flowing, several rapids and small backwaters, basaltic rocks and sand as substrate, riparian vegetation well preserved, urban effluent present.
S9	Ribeirão das Alagoas stream (or Eliezer stream), Eliezer farm, unpaved road at MG427, Conceição das Alagoas, MG	19.58451S; 48.274545W	495	Medium-flow lotic environment; sand and clay as substrate; turbid waters, 1.5 m deep; degraded area
S10	Small stream (no name), unpaved road at a sanitary landfill, tributary of the Uberaba River, Conceição das Alagoas, MG	19.55268S; 48.233689W	507	Lotic environment, low flow, clay soil as a substrate, very shallow water, less than 30 cm deep; few marginal plants, very degraded area
S11	Small stream (no name), 0.7 km at IFTM *campus*, affluent of the Uberaba River, Uberaba, MG	19.67431S; 47.978456W	779	Medium flow stream, gravel and basaltic rocks as substrate; crystalline waters, 1 m deep, dense riparian vegetation
S12	Uberaba River, Carijó farm, 4.5 km upstream from Gorfo waterfall, Conceição das Alagoas, MG	19.92382S; 48.404833W	490	Lotic environment, fast flow, several rapids, basaltic rocks and gravel as a substrate, well preserved riparian vegetation, urban effluent present.
S13	Ribeirão das Alagoas stream (or Eliezer stream), near the confluence with the Uberaba River, Conceição das Alagoas, MG	19.97009S; 48.384722W	506	Lotic environment, medium flow, sand and clay as substrate, large basaltic rocks, turbid water, 1 m depth, degraded riparian vegetation
S14	Small stream (no name), unpaved road at Conceição das Alagoas city, tributary of the Uberaba River, Conceição das Alagoas, MG	19.91363S; 48.375123W	516	Lotic environment, low flow, loam and sand as substrate; shallow water, 70 cm deep; many marginal grasses, degraded area

Specimens were identified using appropriate literature sources (e.g., Langeani et al. 2007; Langeani and Rêgo 2014; Castro et al. 2004; Ota et al. 2018) or by direct comparisons with specimens in museum collections. Vouchers are in the DZSJRP fish collection of the Departamento de Zoologia e Botânica do Instituto de Biociências, Letras e Ciências Exatas, Universidade Estadual Paulista 'Júlio de Mesquita Filho', São José do Rio Preto, SP, Brazil. Some groups are in need of a taxonomic revision, consequently the particle aff. (meaning “not the referred species, but very similar”) is used. The morphometric measurements were taken on the left side of the body, using a digital caliper with an accuracy of 0.01 mm. Lower-level taxonomy and species names follow Fricke et al. (2018) and suprageneric taxonomic groups are those listed in Betancur et al. (2017), except for Cynolebiidae and Bryconidae that follow van der Laan (2016). Allochthonous species are those with their origins from any other hydrographic system in South America outside the Upper Paraná River as defined above. Exotic species are those with origins from any other continent.

## Results

**Figure 3.**
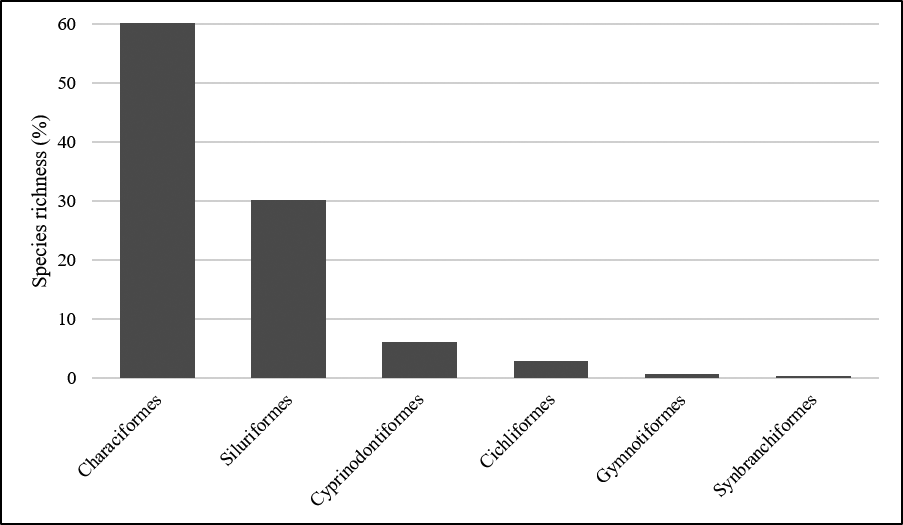
Species richness for each fish order collected in Uberaba River, Upper Paraná River system, Brazil.

**Figure 4.**
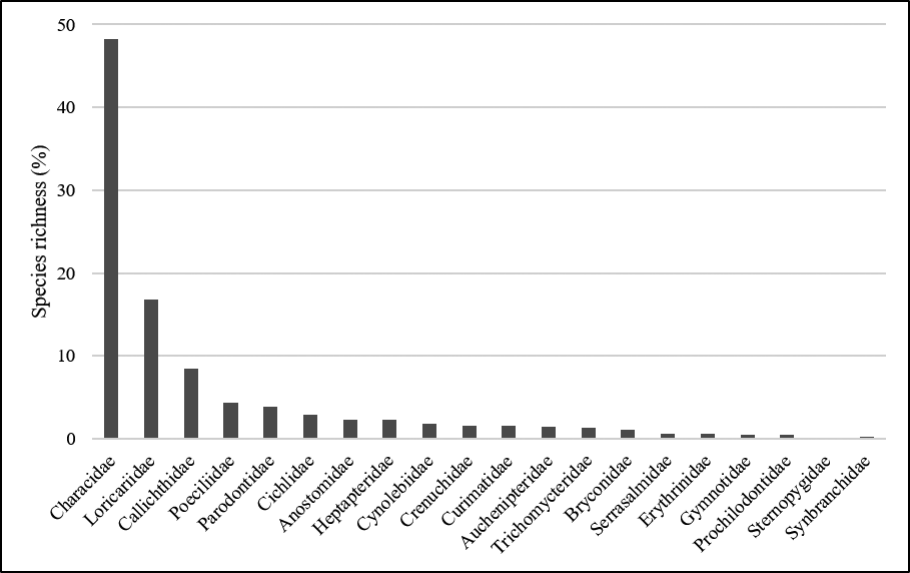
Species richness of each fish family collected in Uberaba River, Upper Paraná River system, Brazil.

**Figure 5.**
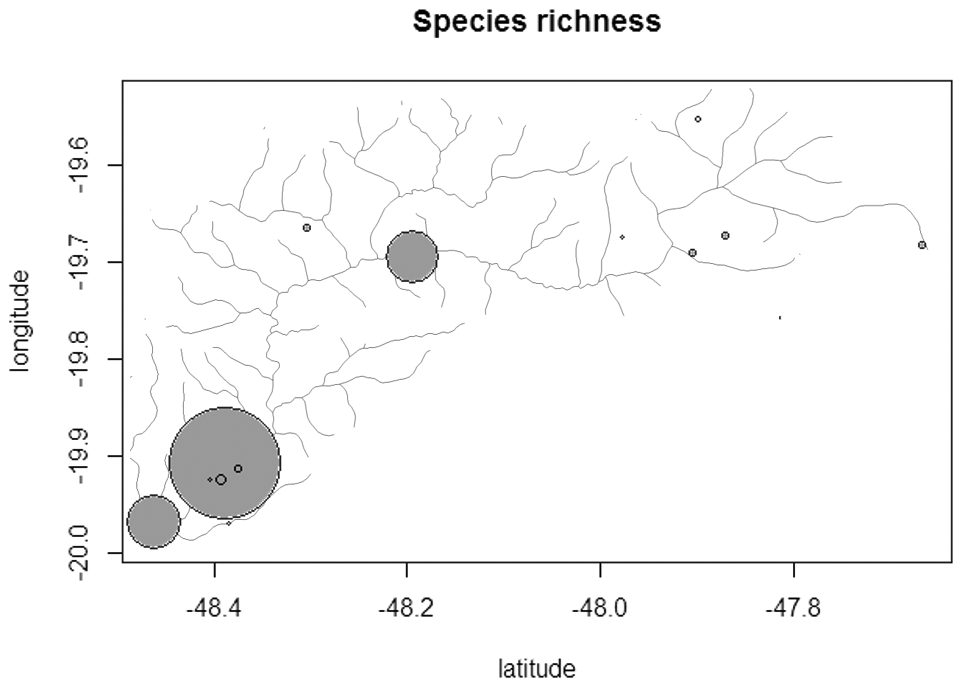
Species richness along longitudinal gradient in Uberaba River, Upper Paraná River system, Brazil. Circle diameter corresponds to species richness.

In total, 2,722 specimens were collected and assigned to 49 genera and 73 species. The identified taxa are listed in Table 2. Most of the species in the Uberaba River are autochthonous (80.0%). Nine species (12.3%) have been recognized as allochthonous (*Galeocharax gulo* (Cope), *Metynnis lippincottianus* (Cope), Knodus aff. moenkhausii (Eigenmann & Kennedy), *Hoplerythrinus unitaeniatus* (Spix & Agassiz), *Gymnotus inaequilabiatus* (Valenciennes), *Trichomycterus brasiliensis* Lütken, *Megalechis thoracata* (Valenciennes), *Poecilia reticulata* Peters, and *Cichla piquiti* Kullander & Ferreira), and only two (2.7%) species are exotic (*Coptodon rendalli* (Boulenger) and *Oreochromis niloticus* (Linnaeus)). Six orders were recognized, of which Characiformes and Siluriformes were the most representative (90.3%), with eight families and 33 species for the former and five families and 27 species for the latter. Gymnotiformes (two families and three spp.), Cichliformes (one family and seven spp.), Cyprinodontiformes (two families and three spp.), and Synbranchiformes (one sp.) together represent 9.7% of the groups collected (Figure 3). Characidae (48.8%) and Loricariidae (16.8%) correspond to the most abundant families (Figure 4) and occur in the entire river system. The species richness suggested a longitudinal gradient, with more species in the lower reaches whereas in the upper reaches the richness does not exceed ten species (Figure 5 and Table 3). The loricariids are mainly represented by *Hypostomus* species, up to 92% of the total loricariid number. The most abundant species is Knodus aff. moenkhausii with 507 collected specimens comprising 38% of all characiform species. All other species have already been recorded in the Upper Paraná River.

**Table 2.** List of fish species from the Uberaba River, Upper Paraná River system, Brazil. Vouchers and origin/status are provided.

Taxa		Voucher	Origin
** CHARACIFORMES **			
** Anostomidae **			
1	*Leporinus amblyrhynchus* Garavello & Britski, 1987	DZSJRP15809	Autochthonous
2	*Leporinus friderici* (Bloch, 1794)	uncataloged	Autochthonous
3	*Leporinus octofasciatus* Steindachner, 1915	DZSJRP16097	Autochthonous
4	*Leporinus striatus* Kner, 1858	DZSJRP21396	Autochthonous
5	*Schizodon nasutus* Kner, 1858	DZSJRP21388	Autochthonous
** Bryconidae **			
6	*Brycon nattereri* Günther, 1864	DZSJRP17489	Autochthonous/VU
** Characidae **			
7	*Astyanax bockmanni* Vari & Castro, 2007	DZSJRP15819	Autochthonous
8	Astyanax aff. fasciatus (Cuvier, 1819)	DZSJRP15818	Autochthonous
9	*Astyanax lacustris* (Lütken, 1875)	DZSJRP21399	Autochthonous
10	Astyanax aff. paranae Eigenmann, 1914	DZSJRP17486	Autochthonous
11	*Astyanax paranae* Eigenmann, 1914	DZSJRP15823	Autochthonous
12	*Bryconamericus turiuba* Langeani et al., 2005	DZSJRP05533	Autochthonous
13	*Galeocharax gulo* (Cope, 1870)	DZSJRP16096	Allochthonous
14	*Hasemania uberaba* Serra & Langeani, 2015	DZSJRP18781	Autochthonous
15	*Hyphessobrycon uaiso* Carvalho & Langeani, 2013	DZSJRP18783	Autochthonous
16	Knodus aff. moenkhausii (Eigenmann & Kennedy, 1903)	DZSJRP15825	Allochthonous
17	*Oligosarcus pintoi* Campos, 1945	DZSJRP05553	Autochthonous
18	*Piabarchus stramineus* (Eigenmann, 1908)	DZSJRP21383	Autochthonous
19	*Piabina argentea* Reinhardt, 1867	DZSJRP17487	Autochthonous
** Serrasalmidae **			
20	*Metynnis lippincottianus* (Cope, 1870)	DZSJRP21397	Allochthonous
21	*Myloplus tiete* (Eigenmann & Norris, 1900)	DZSJRP21398	Autochthonous/EN
22	*Serrasalmus maculatus* Kner, 1858	DZSJRP21386	Autochthonous
** Curimatidae **			
23	*Steindachnerina insculpta* (Fernández-Yépez, 1948)	DZSJRP15812	Autochthonous
** Erythrinidae **			
24	*Hoplerythrinus unitaeniatus* (Spix & Agassiz, 1829)	DZSJRP21402	Allochthonous
25	*Hoplias intermedius* (Günther, 1864)	DZSJRP21389	Autochthonous
26	Hoplias aff. malabaricus (Bloch, 1794)	DZSJRP10546	Autochthonous
** Parodontidae **			
27	*Apareiodon affinis* (Steindachner, 1879)	DZSJRP21391	Autochthonous
28	*Apareiodon ibitiensis* Campos, 1944	DZSJRP15813	Autochthonous
29	*Apareiodon piracicabae* (Eigenmann, 1907)	DZSJRP16100	Autochthonous
30	*Parodon nasus* Kner, 1859	DZSJRP21400	Autochthonous
** Crenuchidae **			
31	Characidium aff. zebra Eigenmann, 1909	DZSJRP17484	Autochthonous
32	Crenuchidae (undescribed genus and species)	DZSJRP15806	Autochthonous
** Prochilodontidae **			
33	*Prochilodus lineatus* (Valenciennes, 1837)	DZSJRP21385	Autochthonous
** GYMNOTIFORMES **			
** Sternopygidae **			
34	*Eigenmannia trilineata* López & Castello, 1966	DZSJRP21392	Autochthonous
** Gymnotidae **			
35	*Gymnotus inaequilabiatus* (Valenciennes, 1839)	uncataloged	Allochthonous
36	*Gymnotus sylvius* Albert & Fernandes-Matioli, 1999	DZSJRP16101	Autochthonous
** SILURIFORMES **			
** Callichthyidae **			
37	*Aspidoras fuscoguttatus* Nijssen & Isbrücker, 1976	DZSJRP18785	Autochthonous
38	*Corydoras difluviatilis* Britto & Castro, 2002	DZSJRP15824	Autochthonous
39	*Megalechis thoracata* (Valenciennes, 1840)	DZSJRP21106	Allochthonous
** Heptapteridae **			
40	*Imparfinis borodini* Mees & Cala, 1989	DZSJRP17488	Autochthonous
41	*Pimelodella avanhandavae* Eigenmann, 1917	DZSJRP21105	Autochthonous
42	*Rhamdia quelen* (Quoy & Gaimard, 1824)	DZSJRP16799	Autochthonous
43	*Rhamdiopsis* sp.	DZSJRP15817	Autochthonous
** Loricariidae **			
44	*Curculionichthys insperatus* (Britski & Garavello, 2003)	DZSJRP21120	Autochthonous
45	*Hypostomus albopunctatus* (Regan, 1908)	DZSJRP21390	Autochthonous
46	*Hypostomus ancistroides* (Ihering, 1911)	DZSJRP15810	Autochthonous
47	*Hypostomus butantanis* (Ihering, 1911)	DZSJRP16098	Autochthonous
48	*Hypostomus fluviatilis* (Schubart, 1964)	DZSJRP21114	Autochthonous
49	Hypostomus aff. hermanni (Ihering, 1905)	DZSJRP21107	Autochthonous
50	*Hypostomus margaritifer* (Regan, 1908)	DZSJRP02107	Autochthonous
51	*Hypostomus nigromaculatus* (Schubart, 1964)	DZSJRP16103	Autochthonous
52	Hypostomus aff. paulinus (Ihering, 1905)	DZSJRP21108	Autochthonous
53	*Hypostomus regani* (Ihering, 1905)	DZSJRP21124	Autochthonous
54	*Hypostomus strigaticeps* (Regan, 1908)	DZSJRP21125	Autochthonous
55	*Hypostomus topavae* (Godoy, 1969)	DZSJRP21098	Autochthonous
56	*Loricaria lentiginosa* Isbrücker, 1979	uncataloged	Autochthonous
57	*Microlepdogaster dimorpha* Martins & Langeani, 2012	DZSJRP18784	Autochthonous
58	*Proloricaria prolixa* (Isbrücker & Nijssen, 1978)	DZSJRP16102	Autochthonous
59	*Rineloricaria latirostris* (Boulenger, 1900)	DZSJRP15811	Autochthonous
** Trichomycteridae **			
60	*Trichomycterus brasiliensis* Lütken, 1874	DZSJRP21116	Allochthonous
61	*Trichomycterus candidus* (Miranda-Ribeiro, 1949)	DZSJRP15820	Autochthonous
** Auchenipteridae **			
62	*Tatia neivai* (Ihering, 1930)	DZSJRP21111	Autochthonous
** CYPRINODONTIFORMES **			
** Cynolebiidae **			
63	*Melanorivulus giarettai* Costa, 2008	DZSJRP18782	Autochthonous
** Poeciliidae **			
64	*Phalloceros harpagos* Lucinda, 2008	DZSJRP17485	Autochthonous
65	*Poecillia reticulata* Peters, 1859	DZSJRP17483	Allochthonous
** CICHLIFORMES **			
** Cichlidae **			
66	*Cichla piquiti* Kullander & Ferreira, 2006	DZSJRP21401	Allochthonous
67	*Cichlasoma paranaense* Kullander, 1983	DZSJRP21394	Autochthonous
68	*Coptodon rendalli* (Boulenger, 1897)	DZSJRP05549	Exotic
69	*Crenicichla britskii* Kullander, 1982	DZSJRP21393	Autochthonous
70	*Crenicichla jaguarensis* Haseman, 1911	DZSJRP21387	Autochthonous
71	*Geophagus brasiliensis* (Quoy & Gaimard, 1824)	DZSJRP21395	Autochthonous
73	*Oreochromis niloticus* (Linnaeus, 1758)	uncataloged	Exotic
** SYNBRANCHIFORMES **			
** Synbranchidae **			
73	*Synbranchus marmoratus* Bloch, 1795	DZSJRP21384	Autochthonous

**Table 3.** Species collected (X) in each site (S1 to S14) of the Uberaba River, Upper Paraná River system, Brazil.

Species	Sites													
	S1	S2	S3	S4	S5	S6	S7	S8	S9	S10	S11	S12	S13	S14
*Apareiodon affinis*							X	X	X			X	X	
*Apareiodon ibitiensis*							X	X	X			X	X	
*Apareiodon piracicabae*								X	X			X	X	
*Aspidoras fuscoguttatus*					X									
*Astyanax bockmanni*								X	X			X	X	
Astyanax aff. fasciatus							X	X	X			X	X	
*Astyanax lacustris*		X		X			X	X	X			X	X	
*Astyanax paranae*			X			X					X			X
Astyanax aff. paranae										X				
*Brycon nattereri*							X	X						
*Bryconamericus turiuba*							X	X						
Characidium aff. zebra									X	X				
*Cichla piquiti*									X					
*Cichlasoma paranaense*								X	X			X	X	
*Coptodon rendalli*							X	X						
*Corydoras difluviatilis*								X				X		
*Crenicichla britskii*								X						
*Crenicichla jaguarensis*								X						
Crenuchidae (undescribed genus and species)	X													
*Curculionichthys insperatus*						X								
*Eigenmannia trilineata*								X						
*Galeocharax gulo*								X						
*Geophagus brasiliensis*							X	X				X	X	
*Gymnotus inaequilabiatus*								X						
*Gymnotus sylvius*								X		X		X		
*Hasemania uberaba*	X													
*Hoplerythrinus unitaeniatus*								X					X	
*Hoplias intermedius*							X	X						
Hoplias aff. malabaricus							X	X				X	X	
*Hyphessobrycon uaiso*	X		X	X										
*Hypostomus albopunctatus*							X	X				X		
*Hypostomus ancistroides*					X			X	X			X	X	
*Hypostomus butantanis*								X				X		
*Hypostomus fluviatilis*								X						
Hypostomus aff. hermani								X				X		
*Hypostomus margaritifer*								X				X		
*Hypostomus nigromaculatus*							X	X	X			X	X	
Hypostomus aff. paulinus							X	X				X		
*Hypostomus regani*								X				X		
*Hypostomus strigaticeps*							X	X				X		
*Hypostomus topavae*							X	X	X			X	X	
*Imparfinis borodini*								X						
Knodus aff. moenkhausii							X	X					X	
*Leporinus amblyrhynchus*							X	X				X		
*Leporinus friderici*							X	X	X				X	
*Leporinus octofasciatus*								X	X				X	
*Leporinus striatus*								X						
*Loricaria lentiginosa*								X				X		
*Megalechis thoracata*													X	X
*Melanorivulus giarettai*	X			X										
*Metynnis lippincottianus*													X	
*Microlepdogaster dimorpha*					X									
*Myloplus tiete*								X					X	
*Oligosarcus pintoi*													X	
*Oreochromis niloticus*							X	X					X	
*Parodon nasus*							X	X	X			X	X	
*Phalloceros harpagos*					X									
*Piabarchus stramineus*							X	X						
*Piabina argentea*		X						X						
*Poecillia reticulata*								X		X		X		
*Prochilodus lineatus*							X							
*Proloricaria prolixa*								X				X		
*Rhamdia quelen*							X	X				X	X	
*Rhamdiopsis* sp.						X								
*Rineloricaria latirostris*								X				X		
*Schizodon nasutus*								X						
*Serrasalmus maculatus*								X						
*Steindachnerina insculpta*							X	X	X			X	X	
*Synbranchus marmoratus*								X		X		X		
*Tatia neivai*								X				X		
*Trichomycterus brasiliensis*											X			
*Trichomycterus candidus*						X								
**Species richness**	**4**	**2**	**2**	**3**	**4**	**4**	**24**	**53**	**15**	**5**	**2**	**31**	**24**	**2**

### Key to fish species of the Uberaba River drainage

**Table:** 

1	Single mid-ventral gill opening; eel-shaped body	***Synbranchus marmoratus***
–	Two laterally located gill openings; not eel-shaped body	**2**
2	Dorsal and pelvic fins absent; anal-fin rays more than 100	**3**
–	Dorsal fin present; pelvic fin commonly present; anal-fin rays up to 50	**5**
3	Body uniformly clear with relatively inconspicuous longitudinal stripes; anal fin not reaching the tail end; terminal mouth, both jaws approximately equal	***Eigenmannia trilineata***
–	Body dark with clear transverse bands; anal fin extending to the tail end; prognathous, lower jaw longer than upper jaw	**4**
4	Obliquely-oriented dark transversal bars fragmented, forming a pattern of irregular spots; anal-fin posterior membrane striped	***Gymnotus inaequilabiatus***
–	Obliquely-oriented dark transversal bars not fragmented; anal-fin posterior region darkly pigmented or translucent	***Gymnotus sylvius***
5	Body naked or covered by bony plates	**6**
–	Body covered by scales	**30**
6	Body covered by bony plates, at least partially	**7**
–	Body covered by thick skin; bony plates absent	**24**
7	Mouth forming a ventral oral disk; bony plates rows on flanks 3–5	**8**
–	Mouth not forming ventral oral disk, with terminal or subterminal opening; bony plates rows on flank 2	**22**
8	Adipose fin absent	**9**
–	Adipose fin present	**12**
9	Caudal peduncle very elongate and depressed	**10**
–	Caudal peduncle rounded or elliptical in cross-section	**13**
10	Lips with small papillae, occasionally with short, thick, non-filamentous projections	***Rineloricaria latirostris***
–	Lips fringed, with filamentous projections	**11**
11	Head with dark brown spots, much smaller than the eye diameter	***Loricaria lentiginosa***
–	Head light brown without spots	***Proloricaria prolixa***
12	Scapular bridge fully exposed; well-developed and pointed odontodes on the anterior portion of the snout	***Curculionichthys insperatus***
–	Scapular bridge exposed only laterally; small and spatulate odontodes on the anterior portion of the snout	***Microlepidogaster dimorpha***
13	Body light with dark spots	**14**
–	Body dark with light spots or vermiculations	**17**
14	Lateral keels on body present (three rows), with hypertrophied odontodes	***Hypostomus ancistroides***
–	Lateral keels on body absent	**15**
15	Pectoral-fin spine claviform, with well-developed odontodes on distal portion; eyes small, 6–6.5 × in head length	***Hypostomus nigromaculatus***
–	Pectoral-fin spine not claviform, with subequal odontodes along entire spine; eyes large, 3.5–5 × in head length	***Hypostomus fluviatilis***
16	Abdomen completely covered by plates; dentary angle more than 60°; bony plates between dorsal and adipose fins 5 pairs	***Hypostomus topavae***
–	Abdomen without plates on pelvic-fin region; dentary angle approximately 45°; bony plates between dorsal and adipose fins 4 pairs	**Hypostomu s aff. hermani**
17	Pectoral-fin spine equal to or shorter than pelvic-fin spine	***Hypostomus albopunctatus***
–	Pectoral-fin spine longer than pelvic-fin spine	**18**
18	Premaxillary and dentary with short and sturdy teeth (18–32), arranged in obtuse angle	**19**
–	Premaxillary and dentary with long and thin teeth (more than 35), arranged in acute angle	**20**
19	Body and fins with light spots, aligned longitudinally, but not forming continuous line	***Hypostomus margaritifer***
–	Head and fins with light vermiculations, with four longitudinal yellow lines on flank, from dorsal fin to caudal-fin base	***Hypostomus butantanis***
20	Pectoral girdle covered with large plates; bony plates between anal and caudal fins 10 or 11; dentary teeth more than 140	**Hypostomus aff. paulinus**
–	Pectoral girdle covered with very small plates or skin; bony plates between anal and caudal fins 12 or 13; dentary teeth up to 130	**21**
21	Mid-lateral plates series 28 or 29; snout-operculum distance greater than the width of the lips; dorsal fin large, reaching adipose fin; premaxillary and dentary teeth more than 65	***Hypostomus regani***
–	Mid-lateral plates series 25 or 26; snout-operculum distance equal to width of the lips; dorsal fin of moderate size, distant from adipose fin; premaxillary and dentary teeth up to 60	***Hypostomus strigaticeps***
22	Mental barbels absent; jaws teeth present; nuchal plate covered by skin; caudal fin truncated	***Megalechis thoracata***
–	Mental barbels present; jaws teeth absent; nuchal plate exposed; caudal fin forked	**23**
23	Supraoccipital long and reaching the nuchal plate; pectoral-fin rays anterior portion without posterior bone lamellae	***Corydoras difluviatilis***
–	Supraoccipital short, not reaching the nuchal plate; pectoral-fin rays anterior portion with posterior bone lamellae (more evident in the first rays)	***Aspidoras fuscoguttatus***
24	Operculum and preoperculum with odontodes; dorsal-fin origin situated posterior the middle of the body	**25**
–	Operculum and preoperculum without odontodes; dorsal-fin origin situated approximately at the middle of the body	**26**
25	Pelvic fin present	***Trichomycterus brasiliensis***
–	Pelvic fin absent	***Trichomycterus candidus***
26	Adipose fin short, shorter than anal fin length; nuchal plate reaching the posterior portion of head	***Tatia neivai***
–	Adipose fin long, approximately 2 × anal fin length; nuchal plate not reaching the posterior portion of head	**27**
27	Body very elongate, depth contained 8.0 × in standard length; 4 dark brown dorsal transverse bands (first at vertical passing at pectoral fin, second at vertical passing anterior portion of dorsal-fin base, third at vertical passing at last third of dorsal-fin base, and the last one at vertical passing at adipose-fin origin); eyes dorsally placed	***Imparfinis borodini***
–	Body short, depth contained up to 6.0 × in standard length; dark brown dorsal transverse bands absent; eyes laterally placed	**28**
28	Body uniformly clear; longitudinal black stripe on flank present; maxillary barbels long, and reaching or surpassing the anal-fin origin	***Pimelodella avanhadavae***
–	Body with small dark spots or irregular vermiculations; longitudinal black stripe on flank absent; maxillary barbels short, never reaching the anal-fin origin	**29**
29	Anal-fin rays up to 12; eyes large, approximately 5 × head length	***Rhamdia quellen***
–	Anal-fin rays more than 15; eyes small, more than 7.5 × head length	***Rhamdiopsis* sp.**
30	Dorsal and anal fins anterior rays modified into spines; pelvic fin in thoracic position, below of pectoral fin; lateral line divided into 2 branches, 1 anterior, near the base of the dorsal fin and another posterior, along the middle portion of the body and caudal peduncle; ctenoid scales	**31**
–	Dorsal and anal fins anterior rays not modified into spines; pelvic fin posteriorly located, close to anal fin; lateral line not divided into 2 branches; cycloid or spinoid scales	**37**
31	Dorsal-fin spines separate from soft rays by notch	***Cichla piquiti***
–	Dorsal-fin spines not separate from soft rays by notch	**32**
32	Body elongate (fusiform), 3.6–5.2 × in standard length; preoperculum posterior margin serrated	**33**
–	Body deep, more than 3.5 × in standard length; preoperculum posterior margin smooth	**34**
33	Scales in longitudinal series 33–40; flank with black transverse bands; dorsal fin with XVI + 14 or 15 rays; anal fin with III + 9 or 10 rays; black humeral blotch present	***Crenicichla britskii***
–	Scales in longitudinal series 41–50; flank without black transverse bands (crossing the longitudinal stripe); dorsal fin with XIX–XXI + 10–12 rays; anal fin with III + 7 or 8 rays; black humeral blotch absent	***Crenicichla jaguarensis***
34	Anterior lateral line with 19 or fewer scales; scales in longitudinal series 22–27; black lateral spot present	**35**
–	Anterior lateral line with 20 or more scales; scales in longitudinal series 28–35; black lateral spot absent	**36**
35	Posterior lateral line with 10–14 scales; scales in longitudinal series 24–27; dorsal fin with XV or XVI + 10–13 rays; black lateral spot on flank larger than the eye diameter	***Geophagus brasiliensis***
–	Posterior lateral line with 5–8 scales; scales in longitudinal series 22 or 23; dorsal fin with XIII or XV + 10–15 rays; black lateral spot approximately equal than the eye diameter	***Cichlasoma paranaense***
36	Scales in transverse series above the lateral line 3 or 3½; gill rakers in inferior branch of the first branchial arch 18 or more	***Oreochromis niloticus***
–	Scales in transverse series above the lateral line 2 or 2½; gill rakers in inferior branch of the first branchial arch 15 or fewer	***Coptodon rendalli***
37	Top of head covered by scales; upper jaw protractile	**38**
–	Top of head not covered by scales; upper jaw non-protractile	**40**
38	Dorsal fin closer to caudal fin than to middle of body; gonopodium absent	***Melanorivulus giarettai***
–	Dorsal fin at middle of body; gonopodium present	**39**
39	Males with intense colored spots in life, black when preserved; females without spots; gonopodium with moderate size (3.2–3.6 × in standard length), with terminal portion almost straight	***Poecilia reticulata***
–	Males and females with vertically elongate black spot on medium portion of flank; gonopodium long (2.6–3.1 × in standard length), with terminal portion trifid and ventrally oriented	***Phalloceros harpagos***
40	Teeth absent in adults	***Steindachnerina insculpta***
–	Teeth present in all life stages	**41**
41	Teeth small, numerous and depressibly implanted in the lips	***Prochilodus lineatus***
–	Teeth well-developed, non-depressibly implanted in the jaw bones	**42**
42	Body fusiform or moderately compressed laterally; abdominal serrae absent	**43**
–	Body very compressed laterally; abdominal serrae present	**70**
43	Teeth incisiform (rabbit-like), truncated or cuspidate, premaxillary and dentary with 3 teeth each, premaxillary with 3 and dentary with 3 or 4 teeth, or premaxillary and dentary with 4 teeth each	**44**
–	Teeth conical or multicuspid, no incisiform; teeth number variable, but not as above	**48**
44	Teeth cuspidate; flank silver in life, spots or bands absent; a conspicuous, horizontally elongate black spot at end of caudal peduncle extending to the median caudal-fin rays	***Schizodon nasutus***
–	Teeth truncated; body with large black spots or longitudinal stripes; horizontally elongate black spot on end of caudal peduncle absent	**45**
45	Premaxillary and dentary with 4 teeth each; 3 large black spots on flank (first bellow dorsal fin, second above the anal-fin base and third at the end of caudal peduncle	***Leporinus friderici***
–	Premaxillary with 3 teeth; dentary with 3 or 4 teeth, body with longitudinal black stripes or transverse bars, large black spots on flank absent	**46**
46	Premaxillary and dentary with 3 teeth each; black longitudinal stripe on flank present; dorsal dark transverse bars (but not reaching the longitudinal stripe) 10 or more; subterminal mouth; prominent snout	***Leporinus amblyrhynchus***
–	Premaxillary with 3 teeth; dentary with 4 teeth; black dorsal transverse bars absent; terminal or subterminal mouth; non-prominent snout	**47**
47	Body elongate, depth 4.1 × in standard length; four longitudinal black stripes on flank; fins usually hyaline or slightly red	***Leporinus striatus***
–	Body deep, depth 3.2 × in standard length; eight black transverse bars on flank; fins yellow, orange or red in life	***Leporinus octofasciatus***
48	Premaxillary teeth in 1 row	**49**
–	Premaxillary teeth in 2 or more rows	**57**
49	Adipose fin absent; posterodorsal portion of head with straight margin; caudal fin rounded or truncate	**50**
–	Adipose fin usually present; posterodorsal portion of head convex or with a posterior projection; caudal fin forked or emarginate	**52**
50	Dorsal-fin rays up to 11; pectoral, pelvic and anal fins without dark brown stripes; teeth canine on maxillary absent	***Hoplerythrinus unitaeniatus***
–	Dorsal-fin rays more than 12; pectoral, pelvic and anal fins with dark brown stripes; teeth canine on maxillary present	**51**
51	Medial margin of dentary bones parallel in ventral view; denticles on tongue absent	***Hoplias intermedius***
–	Medial margin of dentary bones converging towards the symphysis in ventral view; denticles on tongue present	**Hoplias aff. malabaricus**
52	Teeth on anterior portion of dentary absent; lower jaw anterior portion straight	**53**
–	Teeth on anterior portion of dentary present; lower jaw anterior portion rounded	**56**
53	Dentary teeth present	***Parodon nasus***
–	Dentary teeth absent	**54**
54	Black lateral stripe with broad projections above and below, giving a zig-zag appearance; body greenish in life	***Apareiodon ibitiensis***
–	Black lateral stripe without broad projections above and below; 6–8 transverse, rectangular or triangular black thin bars above; body silver in life	**55**
55	Scales in pre-anal series 29 or fewer; premaxillary teeth cusps up to 12	***Apareiodon piracicabae***
–	Scales in pre-anal series 29½ or more; premaxillary teeth cusps 12–15	***Apareiodon affinis***
56	Adipose fin absent; pectoral-fin unbranched rays 10–13; principal caudal-fin rays 16	Crenuchidae (undescribed genus and species)
–	Adipose fin present; pectoral-fin unbranched rays 3; principal caudal-fin rays 18 or 19	**Characidium aff. zebra**
57	Premaxillary teeth in three rows; teeth conical in the symphysis region present	***Brycon nattereri***
–	Premaxillary teeth in two rows; teeth conical in the symphysis region absent	**58**
58	Teeth on the palate present	***Oligosarcus pintoi***
–	Teeth on the palate absent	**59**
59	Anal-fin branched rays more than 30; spinoid scales	***Galeocharax gulo***
–	Anal-fin branched rays up to 29; cycloid scales	**60**
60	Lateral line incomplete	**61**
–	Lateral line complete	**62**
61	Adipose fin present	***Hyphessobrycon uaiso***
–	Adipose fin absent	***Hasemania uberaba***
62	Internal series of premaxillary with 4 teeth; body relatively elongate, depth 3.0–4.2 × in standard length	**63**
–	Internal series of premaxillary with 5 teeth; body relatively deep, depth 1.8–3.6 × in standard length	**66**
63	Upper jaw projecting anteriorly; premaxillary teeth misaligned	***Piabina argentea***
–	Upper and lower jaws of equal size; premaxillary teeth aligned	**64**
64	Supraorbital groove present; caudal-fin lobes covered by small scales	**Knodus aff. moenkhausii**
–	Supraorbital groove absent; scales only at the caudal-fin base	**65**
65	Dorsal stripe broad, extending from the supraoccipital crest to the caudal-fin base, with a gap at the region of the adipose fin; humeral spot conspicuous	***Bryconamericus turiuba***
–	Dorsal stripe narrow, continuous, extending from the supraoccipital crest to the caudal-fin base; humeral spot inconspicuous or absent	***Piabarchus stramineus***
66	Maxillary teeth absent; humeral spot clearly defined, horizontally elongate associated with two diffuse vertical black stripes; fins yellow in life	***Astyanax lacustris***
–	Maxillary teeth present; humeral spot absent or inconspicuous; fins orange or red in life	**67**
67	Flank with a silvery longitudinal stripe; scales on abdomen without chromatophores on distal portion	**Astyanax aff. fasciatus**
–	Flank without silvery longitudinal stripe; scales on abdomen with black chromatophores on distal portion	**68**
68	Body relatively deep, up to 3.0 × in standard length; anal-fin rays 22 or more	***Astyanax bockmanni***
–	Body relatively elongate, more than 3.1 × in standard length; anal-fin rays 20 or fewer	**69**
69	Eye with light iris, silver in life; pelvic-fin tip reaching anal fin	**Astyanax aff. paranae**
–	Eye with dark iris, gold or brown in life; pelvic-fin tip not reaching anal fin	***Astyanax paranae***
70	Teeth tricuspid present; premaxilla and dentary teeth in 1 row	***Serrasalmus maculatus***
–	Teeth tricuspid absent; premaxilla and dentary teeth in 2 rows (the inner dentary row represented by 2 small conical teeth	**71**
71	Adipose-fin base longer than taller; dorsal-fin rays 20 or fewer; pre-dorsal spine present	***Metynnis lippincottianus***
–	Adipose-fin base taller than longer; dorsal-fin rays 20 or more; pre-dorsal spine absent	***Myloplus tiete***

## Discussion

The diversity recorded in the Uberaba River (73) is slightly greater than in similar tributaries of the Grande River in São Paulo state, in which 64 species have been recorded in the tributaries of the Pardo, Turvo, and Sapucaí rivers (Castro et al. 2004). Our data increase the number of species previously recorded for the Uberaba River by 44, which corresponds to an increase of 150% of the species referred so far in the region (see more details in SEMEA 2004; Souza et al. 2016). However, these figures may reflect the differences in sampling methods used by us and the previous authors as well as a larger area investigated in this study. Estimates of species richness and diversity considerably depend on methods used as discussed by Oliveira et al. (2014).

The number of species (73) recorded in the Uberaba River comprises ca. 19% of the total species number known in the Upper Paraná River system when compared to the data in Langeani et al. (2007). The ichthyofauna of the Uberaba River is composed mainly of autochthonous species, few allochthonous species and only two exotic species. The autochthonous origin of some of these species in the Upper Parana River still needs further research. For example, the scarcity of data on the origin or taxonomic status of some putative species such as Knodus aff. moenkhausii, *Trichomycterus brasiliensis* or *Megalechis thoracata*, does not allow to reasonably hypothesize on their origin.

Some species recorded in the Uberaba River potentially correspond to new species and some considerations are provided. *Astyanax fasciatus* (Cuvier) is described for the São Francisco River basin and it is widely distributed in the Paraná-Paraguay drainage and coastal drainages of eastern of Brazil. However, based on the definitions by Eigenmann (1921) it is possible to infer the existence of a "*A. fasciatus* species complex" in the Paraná-Paraguay and other coastal drainages. Thus, the name *A. fasciatus* should be used strictly for the São Francisco River lineage (Melo and Buckup 2006). In the La Plata drainage, the *Hoplias malabaricus* species group is constantly corroborated by morphological, cytogenetic and molecular evidence, and a recognition and taxonomic delineating of new entities is currently in progress (Rosso et al. 2018). Additionally, the nominal species name *Hoplias malabaricus* (Bloch) should be applied exclusively to the Guiana shield lineage (Rosso et al. 2018). Similarly, some authors (see Buckup 1992) suggest that populations of *Characidium zebra* Eigenmann throughout South America represent more than one species. *Characidium zebra* was described in tributaries of the Branco River (Negro River system) in the Amazon. Recent evidence suggests that *C. zebra* populations in the San Francisco and Paraná rivers correspond to the same species (Serrano et al. 2018) distinct from the *C. zebra* populations of the Amazon drainage.

**Figure 6.**
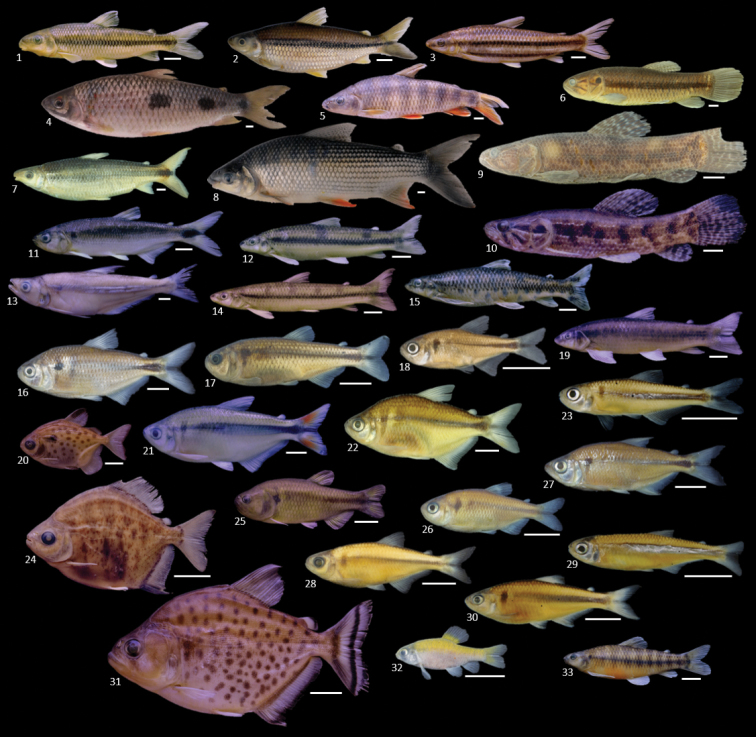
Characiformes collected in the Uberaba River. **1***Leporinus amblyrhynchus***2***Steindachnerina insculpta***3***Leporinus striatus***4***Leporinus friderici* (uncataloged) **5***Leporinus octofasciatus***6***Hoplerythrinus unitaeniatus***7***Schizodon nasutus***8***Prochilodus lineatus***9***Hoplias intermedius***10**Hoplias aff. malabaricus**11***Brycon nattereri***12***Apareiodon piracicabae***13***Galeocharax gulo***14***Apareiodon affinis***15***Apareiodon ibitiensis***16***Astyanax lacustris***17***Astyanax paranae***18**Astyanax aff. paranae**19***Parodon nasus***20***Metynnis lippincottianus***21**Astyanax aff. fasciatus**22***Astyanax bockmanni***23***Bryconamericus turiuba***24***Myloplus tiete***25***Hasemania uberaba***26***Hyphessobrycon uaiso***27***Oligosarcus pintoi***28**Knodus aff. moenkhausii**29***Piabarchus stramineus***30***Piabina argentea***31***Serrasalmus maculatus***32**Crenuchidae (undescribed genus and species) and **33**Characidium aff. zebra. Photographs are of specimens presented in Table 2. Scale bar: 10 mm.

**Figure 7.**
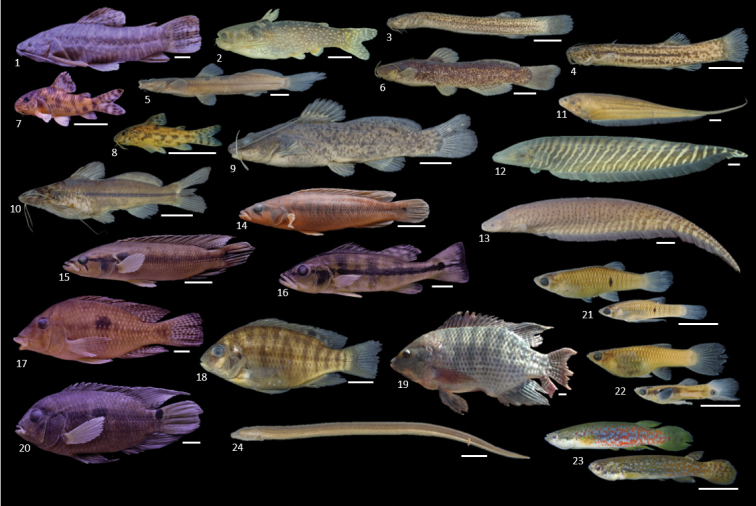
Siluriformes (Loricariidae absent), Gymnotiformes, Cichliformes, Cyprinodontiformes, and Synbrachiformes collected in the Uberaba River. **1***Megalechis thoracata***2***Tatia neivai***3***Trichomycterus candidus***4***Trichomycterus brasiliensis***5***Imparfinis borodini***6***Rhamdiopsis* sp. **7***Corydoras difluviatilis***8***Aspidoras fuscoguttatus***9***Rhamdia quelen***10***Pimelodella avanhadavae***11***Eigenmannia trilineata***12***Gymnotus sylvius***13***Gymnotus inaequilabiatus* (uncataloged) **14***Crenicichla jaguarensis***15***Crenicichla britskii***16***Cichla piquiti***17***Geophagus brasiliensis***18***Coptodon rendalli***19***Oreochromis niloticus* (uncataloged) **20***Cichlasoma paranaense***21***Phalloceros harpagos* (female above and male below) **22***Poecillia reticulata* (female above and male below) **23***Melanorivulus giarettai* (male above and female below) and **24***Synbranchus marmoratus*. Photographs are of specimens presented in Table 2. Scale bar: 10 mm.

Astyanax aff. paranae Eigenmann collected from the Uberaba River may represent a distinct species in the complex “*Astyanax scabripinnis* species complex” sensu Moreira-Filho and Bertollo (1991), a group with an underestimated diversity (Bertaco and Malabarba 2001) as it differs by a number of features (e.g., eye coloration and some measurements). *Knodus moenkhausii* (Eigenmann & Kennedy) was described from the Arroyo Trementina in the Paraguay River system. The specimens from the Upper Paraná River and identified so far as *K. moenkhausii* apparently represents an undescribed species (F. R. Carvalho pers. comm.).

The taxonomic boundaries of the *Hypostomus* species are unclear. Some species of the genus *Hypostomus* are highly variable morphologically and widely distributed. In addition, some important diagnostic characters, such as color pattern, cannot be seen at present in type specimens collected more than 100 years ago, making identification of the species difficult (Zawadzki et al. 2004). For example, *Hypostomus hermanni* Ihering is widely distributed within the Upper Paraná River system. A comparison of the specimens collected in the Uberaba River with specimens from other locations revealed a discrepancy in some meristic and color traits. The Uberaba specimens are especially different from specimens from the Piracicaba River, the type locality of *H. hermanni*. It has been also shown that different populations of *Hypostomus paulinus* (Ihering) are effectively reproductively isolated and characterized by a high degree of inbreeding (Zawadzki et al. 2004).

The occurrence of *Metynnis lippincottianus* may be a result of accidental introduction (Ota 2015). Among the allochthonous species, *Poecilia reticulata* was introduced to control mosquito larvae (Ota et al. 2018). *Cichla piquiti* was probably introduced for sport fishing (Langeani et al. 2007; Ota et al. 2018), and *Gymnotus inaequilabiatus* originally from the Lower Paraná River, Paraguay and Uruguay rivers (Maxime and Albert 2014), colonized the upper reaches of the Paraná River after the construction of the Itaipu hydroelectric dam in the 1980s. Ota et al. (2018) suggested that the occurrence of *Hoplerythrinus unitaeniatus* in the Upper Paraná River can be associated with its introduction as a live bait or after inundation of the Sete Quedas Falls after the construction of the Itaipu dam. *Galeocharax gulo* is widely distributed in almost all Upper Amazon River systems, also in the Orinoco, Oyapok, Araguaia-Tocantins, and Paraná rivers (Giovannetti et al. 2017). The occurrence of this species in the Upper Paraná system may be a result of natural dispersion. *Coptodon rendalli* and *O. niloticus* probably represent results of escapes from fish farms (Langeani et al. 2007; Ota et al. 2018) and the populations of both species are probably established in the region as they have been regularly registered since long ago. Finally, Souza et al. (2016) report the occurrence of *Cyphocharax nagelii* (Steindachner) and *Steindachnerina brevipinna* (Eigenmann & Eigenmann) in the system, but we could not confirm these data and refrained from including them in the species list.

New taxa have been described from the Uberaba River system over the past decade, e.g., *Hasemania uberaba* (Serra and Langeani 2015), *Hyphessobrycon uaiso* (Carvalho and Langeani 2013), and *Microlepidogaster dimorpha* (Martins and Langeani 2011). These newly described species are only known from their type localities or from a few localities corroborating several examples of endemism in the Upper Paraná River, previously indicated by some authors (e.g., Langeani et al. 2007). This clearly demonstrates the importance of inventories and consequent conservation measures. Two species registered in the Uberaba River are definitely threatened: *Brycon nattereri* Günther and *Myloplus tiete* (Eigenmann & Norris) are assigned to “Vulnerable” (VU) and “Endangered” (EN) respectively, on the IBAMA Red List of Endangered Species (ICMBio 2015). The main threats to the local fauna are related to changes in hydrological cycles and the loss of riparian vegetation, as well as overexploitation of natural stocks (Lima et al. 2008; Lima et al. 2015). In addition, the presence of migratory rheophilic species such as *Prochilodus lineatus* (Valenciennes), *Leporinus friderici* (Bloch), *B. nattereri*, and *M. tiete*, is because these species use local resources, at least partially, to complete their life cycle, as suggested by Carolsfeld et al. (2003). Considering all the factors discussed above, the Uberaba River contains a diverse and heterogeneous fish fauna, with two endemic species, *H. uberaba* and an undescribed crenuchid (a description is in the process by Ribeiro et al.) and a low number of allochthonous and exotic species. The Uberaba River has undergone several anthropogenic actions over the last decades, such as the increase of the area destined to grazing, resulting in only 17.7% of native vegetation remains (Valle-Junior et al. 2010) and the reduction of the lotic environments due to damming. The impact of human-induced environmental change is dramatic on the structure and composition of the local fauna. Development of management plans on conservation areas such as the implementation of “Area de Proteção Ambiental Rio Uberaba – APA-Rio Uberaba” project (SEMEA 2004) is necessary to mitigate the effects and help the sustainable use of local natural resources.

**Figure 8.**
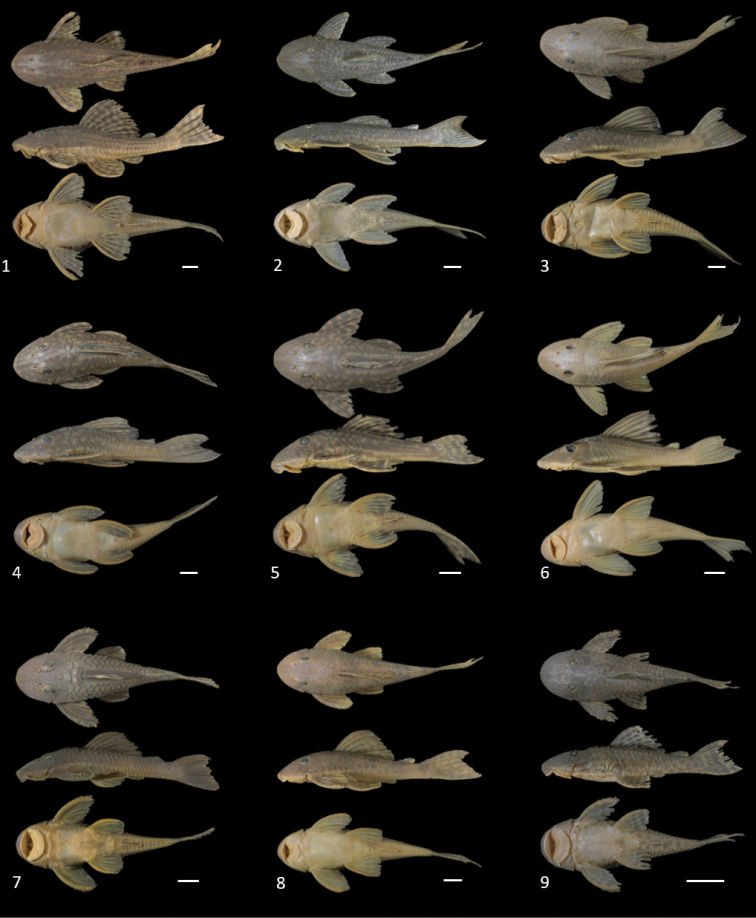
Loricariidae, genus *Hypostomus* collected in the Uberaba River (dorsal, lateral, and ventral photographs). **1***Hypostomus ancistroides***2***Hypostomus albopunctatus***3***Hypostomus strigaticeps***4***Hypostomus margaritifer***5***Hypostomus butantanis***6***Hypostomus regani***7**Hypostomus aff. paulinus**8***Hypostomus topavae* and **9***Hypostomus nigromaculatus*, Photographs are of specimens presented in Table 2. Scale bar: 10 mm.

**Figure 9.**
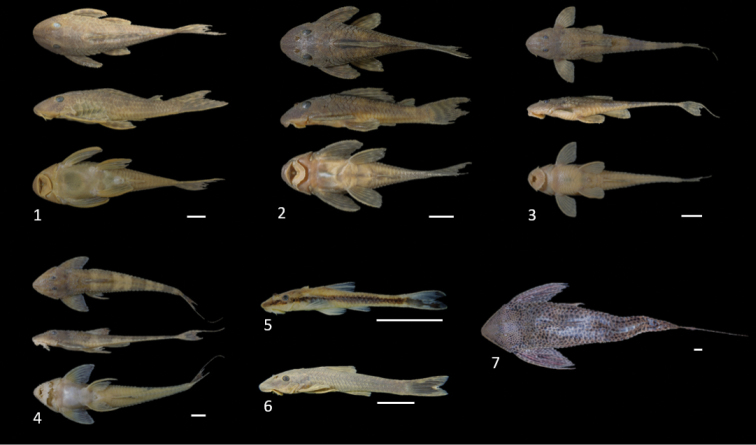
Another loricariids collected in the Uberaba River (dorsal, lateral, and ventral photographs). **1**Hypostomus aff. hermanni**2***Hypostomus fluviatilis***3***Rineloricaria latirostris***4***Proloricaria prolixa***5***Curculionichthys insperatus***6***Microlepdogaster dimorpha* and **7***Loricaria lentiginosa* (uncataloged). Photographs are of specimens presented in Table 2. Scale bar: 10 mm.
